# Changes in Adiposity and Cerebrospinal Fluid Biomarkers Following a Modified Mediterranean Ketogenic Diet in Older Adults at Risk for Alzheimer’s Disease

**DOI:** 10.3389/fnins.2022.906539

**Published:** 2022-06-02

**Authors:** Tina E. Brinkley, Iris Leng, Thomas C. Register, Bryan J. Neth, Henrik Zetterberg, Kaj Blennow, Suzanne Craft

**Affiliations:** ^1^Department of Internal Medicine, Section on Gerontology and Geriatric Medicine, Winston-Salem, NC, United States; ^2^Division of Public Health Sciences, Department of Biostatistics and Data Sciences, Winston-Salem, NC, United States; ^3^Department of Pathology/Comparative Medicine, Wake Forest School of Medicine, Winston-Salem, NC, United States; ^4^Department of Neurology, Mayo Clinic, Rochester, MN, United States; ^5^Department of Psychiatry and Neurochemistry, University of Gothenburg, Gothenburg, Sweden; ^6^Clinical Neurochemistry Laboratory, Sahlgrenska University Hospital, Gothenburg, Sweden; ^7^Department of Neurodegenerative Disease, UCL Queen Square Institute of Neurology, London, United Kingdom; ^8^United Kingdom Dementia Research Institute at UCL, London, United Kingdom; ^9^Hong Kong Center for Neurodegenerative Diseases, Hong Kong, Hong Kong SAR, China

**Keywords:** ketogenic diet, adiposity, CSF biomarkers, mild cognitive impairment, subjective memory complaints, prediabetes

## Abstract

**Background:**

Ketogenic diets have been used to treat both obesity and neurological disorders, including epilepsy and more recently Alzheimer’s disease (AD), likely due to favorable effects on both central and peripheral metabolism. Improvements in body composition have also been reported; however, it is unclear if diet-induced changes in adiposity are related to improvements in AD and related neuropathology.

**Purpose:**

We examined the effects of a Modified Mediterranean Ketogenic (MMK) diet vs. an American Heart Association (AHA) diet on body weight, body composition, and body fat distribution and their association with cerebrospinal fluid (CSF) biomarkers in older adults at risk for AD.

**Methods:**

Twenty adults (mean age: 64.3 ± 6.3 years, 35% Black, 75% female) were randomly assigned to a crossover trial starting with either the MMK or AHA diet for 6 weeks, followed by a 6-week washout and then the opposite diet for 6 weeks. At baseline and after each diet adiposity was assessed by dual-energy x-ray absorptiometry and CSF biomarkers were measured. Linear mixed effect models were used to examine the effect of diet on adiposity. Spearman correlations were examined to assess associations between adiposity and CSF biomarkers.

**Results:**

At baseline there was a high prevalence of overweight/obesity and central adiposity, and higher visceral fat and lower peripheral fat were associated with an adverse CSF biomarker profile. The MMK and AHA diets led to similar improvements in body composition and body fat distribution. Significant correlations were found between changes in adiposity and changes in CSF biomarkers (r’s = 0.63–0.92, p’s < 0.05), with notable differences by diet. Decreases in body fat on the MMK diet were related to changes in Aβ biomarkers, whereas decreases in body fat on the AHA diet were related to changes in tau biomarkers and cholinesterase activity. Interestingly, increases in CSF Aβ on the MMK diet occurred in those with less fat loss.

**Conclusion:**

An MMK diet leads to favorable changes in body composition, body fat distribution, and CSF biomarkers. Our data suggest that modest weight loss that maximizes visceral fat loss and preserves peripheral fat, may have the greatest impact on brain health.

**Clinical Trial Registration:**

[www.ClinicalTrials.gov], identifier [NCT02984540].

## Introduction

Overweight and obesity are associated with adverse changes in brain structure and function (Gustafson et al., 2004a,b; Ward et al., 2005) and an increased risk for Alzheimer’s disease (AD) in middle age (Emmerzaal et al., 2015). Studies show that mid-life obesity increases risk for AD by as much as threefold (Kivipelto et al., 2005; Hassing et al., 2009; Xu et al., 2011), and for every 1-unit increase in mid-life body mass index (BMI), the risk of AD increases by ∼7% and the age of AD onset decreases by ∼6.7 months (Chuang et al., 2016). Higher BMI in mid-life is also associated with neuropathological hallmarks of AD, i.e., greater β-amyloid (Aβ) and tau deposition (Mrak, 2009; Chuang et al., 2016; Merrill et al., 2016). The opposite is true in late life (Ewers et al., 2012; Hsu et al., 2016; Alosco et al., 2017) when the relationship between obesity and AD is attenuated or reversed, and lower body weight or weight loss portends a worse prognosis (Luchsinger et al., 2007; Atti et al., 2008; Fitzpatrick et al., 2009; West and Haan, 2009; Tolppanen et al., 2014).

Age-related changes in body composition and body fat distribution likely contribute to late-life changes in the relationship between obesity and AD. Increases in fat mass, particularly in central (i.e., trunk, abdominal) and visceral fat depots, are common with advancing age, even in the absence of significant changes in BMI (Zamboni et al., 2003). Visceral adiposity is a well-established risk factor for cardiometabolic disease; on the other hand, accumulation of subcutaneous fat in peripheral depots (i.e., hip and thighs) may confer protection against metabolic dysfunction and related diseases (Lee et al., 2013). Thus, anthropometric measures of adiposity such as BMI and waist circumference, which cannot distinguish the location of excess fat, may not be sufficient to fully understand the associated risks for AD, particularly in older adults (West and Haan, 2009). Despite growing evidence that visceral and peripheral adiposity may have significant, and likely distinct, effects on brain health (Forte et al., 2017; Pflanz et al., 2022), no studies have examined their relationship with specific measures of AD neuropathology.

Interventions that can modulate pathways involved in both AD and obesity may provide additional insight into the underlying mechanisms linking these complex diseases. The ketogenic diet, characterized by low carbohydrate and high fat intake (Maalouf et al., 2009), has been shown to reduce Aβ pathology (Van der Auwera et al., 2005), improve memory (Krikorian et al., 2012), and enhance weight loss and body composition (Amini et al., 2021). However, the joint effects of a ketogenic diet on AD biomarkers and adiposity have not been studied to date. We recently reported that a Modified Mediterranean Ketogenic (MMK) was associated with increases in cerebrospinal fluid (CSF) Aβ42 and decreases in tau, as well as reductions in CSF neurofilament light (NFL), neurogranin, and soluble triggering receptor expressed on myeloid cells 2 (sTREM) in older adults at risk for AD (Neth et al., 2020). The control diet also led to significant decreases in CSF tau levels; however, there was no significant change in any other CSF biomarker. In the present study, we further investigated the effects of the MMK diet on body composition and body fat distribution and examined their association with multiple CSF biomarkers of AD pathology.

## Materials and Methods

### Study Participants

Community-dwelling adults at risk for AD dementia based on the presence of prediabetes and either subjective memory complaints (SMC) or amnestic mild cognitive impairment (MCI) were recruited to participate. Pre-diabetes was defined according to American Diabetes Association guidelines as a hemoglobin A1c level between 5.7% and 6.4% (American Diabetes Association, 2015). SMC were defined according to Alzheimer’s Disease Neuroimaging Initiative criteria as a Cognitive Change Index score ≥ 16 on the first 12 items (Risacher et al., 2015). MCI was diagnosed by expert physicians and neuropsychologists using National Institute on Aging and Alzheimer’s Association criteria (Albert et al., 2011). Individuals with a prior diagnosis of neurological or neurodegenerative illness (except MCI), major psychiatric disorder, stroke, use of diabetes and lipid-lowering medications, or use of medications with known central nervous system effects including anti-seizure medications, anti-psychotics, and opioids were excluded. The protocol was approved by the Wake Forest Institutional Review Board (ClinicalTrials.gov Identifier: NCT02984540), and all participants and/or their study partners provided written informed consent prior to participating.

### Study Design and Procedures

The study consisted of a randomized crossover design in which participants consumed either the MMK diet or an American Heart Association (AHA) low-fat diet (Krauss et al., 2001) for 6 weeks, followed by a 6-week washout period in which participants were instructed to resume their pre-study diet. Afterward the second diet was consumed for 6 weeks. Lumbar punctures (LPs) and dual-energy x-ray absorptiometry (DXA) scans were completed prior to randomization and at the end of each diet. Adherence and blood metabolic laboratory profiles were also assessed at the half-way point of each diet period. Participants completed a 3-day record in which they recorded all food consumed on 2 weekdays and 1 weekend day at baseline and during weeks 3 and 6 of the diet interventions. Total calorie and macronutrient intakes were calculated from the food records using ProNutra software (Viocare, Princeton, NJ, United States).

### Dietary Interventions

The two dietary interventions were isocaloric, with the main distinction being the proportion of calories from carbohydrates and fat. Specifically, the target macronutrient composition (expressed as % of total calories) for the MMK diet was approximately 5–10% carbohydrate, 60–65% fat, and 30% protein, whereas the target macronutrient composition for the AHA diet was 55–65% carbohydrate, 15–20% fat, and 20–30% protein. Participants worked closely with a registered dietitian (RD) to keep macronutrients within the desired target ranges. The RD developed daily meal plans, a food list, and other educational material for each study participant based on the 3-day food diary, activity level, and macronutrient requirements to help the participants make appropriate food purchases. During the MMK diet period, participants were encouraged to consume healthy fats, protein sources low in saturated fat (i.e., fish, lean meats), fruits, vegetables, and whole grains (within limits), while avoiding low-carbohydrate store-bought products and artificially sweetened beverages. Participants were supplied with 1 L of extra-virgin olive oil to use as the primary source of healthy fat in their diet and were allowed one glass of wine per day. During the AHA diet period, participants were encouraged to eat plentiful fruits, vegetables, and carbohydrates containing adequate fiber, as well as proteins from healthy sources such as fish and lean meat, while limiting their fat intake to < 40 g/d (with no provision of supplementary olive oil). To minimize potential confounding effects on metabolism, participants were asked to keep their exercise and physical activity levels stable and to discontinue the following supplements: resveratrol, coenzyme Q10, coconut oil/other medium chain triglyceride-containing (i.e., Axona) supplements, and curcumin. However, they were provided a multivitamin (Centrum Silver) to use daily throughout the diet period. To monitor adherence, food records and capillary ketone measurements were reviewed during weekly in-person and phone-based visits with the study RD. As previously reported, adherence to the diets was high (≥ 90%) resulting in the expected changes in macronutrient composition for both diets, as well as increases in fasting ketone body levels on the MMK diet (Neth et al., 2020).

### Cerebrospinal Fluid Biomarkers

LPs were conducted after a 12-h overnight fast at baseline and at the end of each diet period. Following local anesthesia with 1% lidocaine, a 22-gauge Sprotte needle was used to collect CSF from the L3-4 or L4-5 interspace while participants rested in the seated or lateral decubitus position. CSF was transferred into pre-chilled polypropylene tubes, frozen immediately on dry ice, and stored at −80°C until analysis of key biomarkers. Aβ42, total tau, and phospho-tau (tau-p181) were measured using a Luminex-based INNO-BIA AlzBio3 assay. Aβ40 was measured using a standard enzyme-linked immunosorbent assay according to manufacturer instructions (Fujirebio, Malvern, PA, United States). Other emerging AD biomarkers in CSF including neurogranin, sTREM2 (Meso Scale Discovery), YKL-40 (R&D Systems, Minneapolis, MN, United States), and NFL (Uman-Diagnostics, Umeå, Sweden) were measured using in-house or commercially-available assays, as previously described (Portelius et al., 2018; Gisslén et al., 2019; Neth et al., 2020). Acetylcholinesterase (AChE) and butyrylcholinesterase (BChE) activities were measured as previously described (Parnetti et al., 2011). All assays were performed by board-certified laboratory technicians blinded to clinical data. Baseline and post-diet samples were analyzed side-by-side on the same assay plates, and intra-assay coefficients of variation were below 10%.

### Body Composition and Fat Distribution

Whole body DXA scans were performed on a Hologic Horizon scanner (Hologic Inc., Belford, MA, United States) using the BodyLogic scan and APEX analysis software (version 5.5.3.1) to determine whole body and regional measures of fat and lean tissue. Regional boundaries were demarcated automatically using anatomical landmarks. The trunk region includes the neck, chest, abdominal and pelvic areas. The leg region includes all the area below the trunk. The android (waist) region is the area between the ribs and the pelvis and is totally enclosed by the trunk region. The gynoid (hip) region includes the hips and upper thighs and overlaps both the leg and trunk regions. Regional adiposity is reported as both absolute fat mass and the percentage of fat in each region. Visceral and subcutaneous adipose tissue (VAT, SAT) cross-sectional areas in the abdominal region were estimated using InnerCore VAT Assessment technology. Measures of fat distribution including trunk/leg, android/gynoid, and VAT/SAT ratios were calculated.

### Statistical Analyses

Baseline characteristics were described as mean (standard deviation) or N (%) overall and by cognitive group (SMC vs. MCI). Linear mixed effect models were fitted separately for the MMK and AHA diets to examine the effect of diet on adiposity. Time (pre vs. post) was the repeated factor, with cognitive group as an independent factor. Age and diet order (MMK diet first vs. AHA diet first) were included as covariates. Interactions between time and cognitive group and diet order were examined. Diet carryover effects and diet effects were examined in the framework of crossover design using the Grizzle two-stage model. Spearman correlations were used to examine cross-sectional and longitudinal associations between adiposity and CSF biomarkers. All analyses were done using SAS 9.4 (Cary, NC, United States).

## Results

### Study Population

The present analysis includes all 20 participants from the parent study. As shown in Table 1, and reported previously in the main paper (Neth et al., 2020), the study population was on average 64 years of age (range: 55–84 years), well-educated (mean years of education: 16.1), and predominately female (75%). Over one-third of participants were Black (35%), 32% were carriers of the *APOE* ε4 allele, and 9 (45%) had MCI. In addition, most participants had increased adiposity as shown by the high prevalence of overweight/obesity, high waist circumference, and increased VAT deposition. There were no statistically significant differences in demographics between the SMC and MCI groups at baseline, although the proportions of Black participants and *APOE* ε4 carriers, as well as the prevalence of obesity and high VAT area were two- to threefold greater in the MCI group. Baseline CSF biomarker levels were also similar between groups.

**TABLE 1:** Baseline characteristics overall and stratified by cognitive group.

Variable	Overall (*n* = 20)	SMC (*n* = 11)	MCI (*n* = 9)
**Demographics**			
Age (years)	64.3 ± 6.3	64.9 ± 7.89	63.4 ± 4.0
Female sex	15 (75.0%)	9 (81.8%)	6 (66.7%)
Black participants	7 (35.0%)	2 (18.2%)	5 (55.6%)
Education (years)	16.1 ± 2.5	16.5 ± 2.3	15.7 ± 2.9
*APOE* ε4 carrier	6 (31.6%)	2 (20.0%)	4 (44.4%)
MMSE score (0–30)	28.7 ± 1.1	28.9 ± 1.0	28.3 ± 1.2
**Adiposity#**			
Overweight	7 (35.0%)	4 (36.4%)	3 (33.3%)
Obese	7 (35.0%)	2 (18.2%)	5 (55.6%)
High waist circumference	15 (79.0%)	9 (81.8%)	6 (75.0%)
High VAT area	11 (58.0%)	4 (36.4%)	7 (87.5%)
**CSF Biomarkers**			
Aβ40 (pg/ml)	10,070 ± 5,215	11,857 ± 5,244	7,517 ± 4,285
Aβ42 (pg/ml)	308 ± 67	316 ± 76	296 ± 57
Aβ42/40 ratio	0.04 ± 0.03	0.03 ± 0.02	0.06 ± 0.04
tau (pg/ml)	38 ± 26	35 ± 26	42 ± 26
tau-p181 (pg/ml)	35 ± 15	34 ± 17	35 ± 12
tau-p181/tau ratio	1.13 ± 0.50	1.23 ± 0.56	0.99 ± 0.41
Aβ42/tau-p181 ratio	9.78 ± 2.90	10.30 ± 3.04	9.00 ± 2.74
NFL (ng/l)	535 ± 247	556 ± 268	488 ± 218
Neurogranin (pg/ml)	110 ± 135	127 ± 153	64 ± 68
YKL-40 (ng/ml)	137 ± 40	135 ± 420	140 ± 423
AChE (U/l)	56 ± 13	57 ± 12	54 ± 17
BChE (U/l)	29 ± 7	30 ± 5	28 ± 10
AChE/BChE	1.94 ± 0.15	1.92 ± 0.16	1.99 ± 0.14
sTREM2 (ng/ml)	4.1 ± 2.1	4.4 ± 2.0	3.3 ± 2.5

*Table values are mean ± SD or N (%).Sample sizes are n = 19–20 for the demographic and adiposity variables and n = 13–17 for the CSF biomarkers.#Overweight defined as BMI = 25.0–29.9 kg/m^2^; Obese defined as BMI > 30 kg/m^2^; High waist circumference defined as > 102 cm (40 in) in men or > 88 cm (35 in) in women; High VAT area defined as > 100 cm^2^.*

### Intervention Effects on Adiposity

Table 2 shows the effects of the MMK and AHA diets on body weight, body composition, and body fat distribution using linear mixed models adjusted for age, baseline cognitive group (SMC vs. MCI), and diet order. There were significant intervention effects for both diets, with reductions in all adiposity variables except the VAT/SAT ratio. Results were similar when models included only the participants who were assigned to the MMK diet or AHA diet first. Based on the two-stage mode, there were no significant differential carryover effects (p’s > 0.20). Although the magnitude of improvement in adiposity was similar between diets, body weight (*p* = 0.03), BMI (*p* = 0.02), total lean mass (*p* = 0.003), and android/gynoid ratio (*p* = 0.03) were lower after the MMK diet compared to the AHA diet.

**TABLE 2:** Effect of MMK and AHA diets on body weight, body composition and body fat distribution.

Variable		MMK Diet		AHA Diet		
	**Baseline**	**Post diet**	**Time effect**	**Post diet**	**Time effect**	**Diet effect#**
Body weight	79.1 ± 16.9	73.2 (3.4)	−5.95 (0.82)*	74.8 (3.4)	−4.4 (0.6)*	−3.8 (1.2)φ
BMI (kg/m^2^)	28.4 ± 5.8	26.3 (1.3)	−2.07 (0.26)*	26.8 (1.2)	−1.5 (0.2)*	−1.3 (0.5)φ
Waist circumference (cm)	105.4 ± 15.7	101.8 (3.8)	−3.8 (1.0)ǂ	102.4 (3.7)	−3.2 (1.3) φ	−2.0 (1.8)
Total fat mass (kg)	31.4 ± 10.7	28.2 (2.5)	−3.2 (0.4)*	28.5 (2.5)	−2.9 (0.4)*	−1.1 (0.9)
Total fat (%)	39.0 ± 8.2	37.5 (2.1)	−1.47 (0.28)*	37.3 (2.0)	−1.7 (0.3)*	0.2 (0.5)
Total lean mass (kg)	45.9 ± 10.2	43.7 (2.0)	−2.3 (0.4)*	44.6 (2.0)	−1.3 (0.3)ǂ	−2.1 (0.6)ǂ
Total lean (%)	58.1 ± 7.8	59.3 (2.0)	1.2 (0.3)ǂ	59.6 (1.9)	1.6 (0.3)*	−0.4 (0.5)
Trunk fat mass (kg)	14.9 ± 5.9	13.1 (1.3)	−1.8 (0.3)*	13.4 (1.3)	−1.6 (0.2)*	−0.7 (0.4)
Trunk fat (%)	37.5 ± 8.7	35.6 (2.2)	−1.9 (0.3)*	35.5 (2.2)	−2.0 (0.4)ǂ	−0.02 (0.7)
Leg fat mass (kg)	11.6 ± 4.1	10.6 (1.0)	−1.0 (0.2)*	10.7 (1.0.)	−0.9 (0.2)*	−0.3 (0.3)
Leg fat (%)	42.0 ± 10.3	40.9 (2.5)	−1.1 (0.3)ǂ	40.5 (2.4)	−1.5 (0.4)ǂ	0.3 (0.5)
Trunk/leg ratio	0.92 ± 0.21	0.89 (0.05)	−0.03 (0.01)ǂ	0.90 (0.05)	−0.03 (0.01) φ	−0.0 (0.01)
Android fat mass (kg)	2.6 ± 1.3	2.2 (0.3)	−0.4 (0.1)*	2.3 (0.3)	−0.3 (0.0)*	−0.2 (0.1)
Android fat (%)	38.9 ± 9.2	36.0 (2.4)	−2.9 (0.6)*	36.3 (2.3)	−2.6 (0.5)*	−1.0 (1.1)
Gynoid fat mass (kg)	5.4 ± 1.6	4.8 (0.4)	−0.5 (0.1)*	4.9 (0.4)	−0.5 (0.1)*	−0.2 (0.1)
Gynoid fat (%)	41.7 ± 8.7	40.5 (2.1)	−1.2 (0.4)ǂ	40.0 (2.1)	−1.7 (0.4)*	0.7 (0.5)
Android/gynoid ratio	0.95 ± 0.23	0.90 (0.05)	−0.06 (0.01)ǂ	0.92 (0.05)	−0.03 (0.01) φ	−0.05 (0.02)φ
VAT area (cm^2^)	123.9 ± 62.5	105.1 (14.3)	−19.0 (4.6)ǂ	106.6 (14.0)	−17.5 (3.2)*	−4.7 (6.2)
SAT area (cm^2^)	376.5 ± 169.0	329.3 (41.4)	−48.4 (6.5)*	338.7 (40.7)	−38.7 (5.4)*	−25.3 (13.1)
VAT/SAT ratio	0.35 ± 0.16	0.36 (0.04)	0.00 (0.01)	0.35 (0.04)	−0.00 (0.01)	0.01 (0.02)

Baseline values are mean ± SD. Post diet values and time and diet effects are reported as least square mean (SE) and β (SE), respectively, adjusted for age, diet order, and cognitive group.*p ≤ 0.0001, ǂ p ≤ 0.01, ^φ^p ≤ 0.05. ^#^Difference between diets, MMK – AHA.Sample sizes are n = 19 for VAT area, SAT area, and VAT/SAT ratio and n = 20 for all other variables.

Time × diet order interactions were not statistically significant; however, significant time × cognitive group interactions were found for total lean mass for both the MMK diet (*p* = 0.0002) and the AHA diet (*p* = 0.003). In addition, significant interactions were found for body weight (*p* = 0.007) and BMI (*p* = 0.01) for the MMK diet only. These results showed that the MCI group had greater reductions in body weight, BMI, and total lean mass compared to the SMC group. Further analyses revealed that the MCI group was heavier (87.8 ± 13.0 vs. 72.1 ± 16.9 kg, *p* = 0.03) and had a higher total lean mass (51.5 ± 9.5 kg vs. 41.3 ± 8.6 kg, *p* = 0.02) at baseline compared to the SMC group. However, baseline BMI (30.2 ± 4.7 kg/m^2^ vs. 26.9 ± 6.3 kg/m^2^, *p* = 0.22) and total % lean (58.5 ± 7.0% vs. 57.8 ± 8.8%, *p* = 0.85) were similar between groups.

### Cross-Sectional Associations Between Adiposity and Cerebrospinal Fluid Biomarkers at Baseline

At baseline, a higher VAT/SAT ratio was correlated with higher NFL (*r* = 0.73, *p* = 0.0065), neurogranin (*r* = 0.79, *p* = 0.0061), sTREM2 (*r* = 0.63, *p* = 0.026), AChE (*r* = 0.75, *p* = 0.0051), and BChE (*r* = 0.73, *p* = 0.0065). Significant correlations were also found between NFL and total fat mass (*r* = −0.55, *p* = 0.049), total % lean (*r* = 0.56, *p* = 0.046), and leg fat mass (*r* = −0.60, *p* = 0.031). Both AChE and BChE were inversely correlated with leg fat mass (*r* = −0.62, *p* = 0.025 and *r* = −0.60, *p* = 0.031, respectively) and gynoid fat mass (*r* = −0.55, *p* = 0.049 and *r* = −0.58, *p* = 0.039, respectively). There were no statistically significant associations between CSF amyloid or CSF tau and any of the adiposity variables at baseline; however, higher CSF tau-p181 was correlated with higher VAT area (*r* = 0.52, *p* = 0.059) and a higher VAT/SAT ratio (*r* = 0.48, *p* = 0.08). Higher VAT was also correlated with a lower CSF Aβ42/tau-p181 ratio (*r* = −0.49, *p* = 0.075).

### Associations Between Changes in Adiposity and Cerebrospinal Fluid Biomarkers on the Modified Mediterranean Ketogenic Diet

CSF biomarker levels following the MMK diet can be found in Supplementary Table 1. Changes in CSF Aβ42 and the CSF Aβ42/Aβ40 ratio in response to the MMK diet were positively associated with changes in leg fat, gynoid fat and android fat (r’s = 0.52–0.68, p’s < 0.05), as shown in Table 3. Changes in the CSF Aβ42/Aβ40 ratio were also positively associated with changes in waist circumference (*r* = 0.58, *p* = 0.037), total % fat (*r* = 0.71, *p* = 0.0045), and trunk % fat (*r* = 0.66, *p* = 0.011). Additionally, changes in the CSF Aβ42/tau-p181 ratio were positively correlated with changes in android % fat (*r* = 0.53, *p* = 0.049), whereas changes in the tau-p181/tau ratio were inversely associated with changes in the android/gynoid ratio (*r* = −0.58, *p* = 0.039). Weight loss was positively associated with reductions in YKL-40 (*r* = 0.62, *p* = 0.023) and BChE activity (*r* = 0.61, *p* = 0.0028), and a strong positive correlation was found between changes in neurogranin and changes in VAT area (*r* = 0.89, *p* = 0.0005). Representative plots are shown in Supplementary Figure 1, which indicate an outlying data point. After removing this data point in sensitivity analyses, the correlations remained signficant (r’s = 0.62–0.64, p’s < 0.05).

**TABLE 3:** Correlations between changes in adiposity and CSF biomarkers on the MMK and AHA diets.

	Aβ42	Aβ42/40	tau	p-tau	Aβ42/tau-p181	p-tau/tau	NG	YKL-40	AChE	BChE	AChE/BChE
Body weight								0.62φ		0.61φ	
BMI								0.66φ			
Waist Circumference		0.58φ							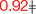		
Total fat Mass %		0.71ǂ	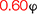			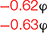					
Total lean Mass %									0.66φ		
Trunk fat Mass %		0.66φ				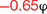		0.63φ			
Leg fat Mass %	0.53φ	0.68ǂ	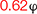						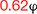		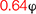
Android fat Mass %	0.52φ	0.61φ			0.53φ					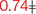	
Gynoid fat Mass %	0.60φ 0.63φ	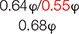		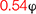		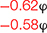					
Android/gynoid ratio						−0.58φ				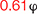	
VAT area							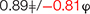				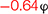
VAT/SAT ratio											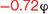

*Table values are Spearman correlation coefficients for the MMK diet (shown in black) and the AHA diet (shown in red),ǂp ≤ 0.01, φp ≤ 0.05.Sample sizes are n = 8–14.*

### Associations Between Adiposity and Cerebrospinal Fluid Biomarkers on the American Heart Association Diet

CSF biomarker levels following the AHA diet are shown in Supplementary Table 1. Changes in the CSF Aβ42/Aβ40 ratio in response to the AHA diet were positively correlated with changes in gynoid fat (*r* = 0.55, *p* = 0.041) (Table 3). Changes in CSF tau and tau-p181 were positively correlated with changes in total fat mass and leg fat mass and gynoid % fat, respectively (r’s = 0.54–0.62, p’s < 0.05), whereas changes in the CSF tau-p181/tau ratio were inversely correlated with changes in total fat, trunk fat, and gynoid fat (r’s = −0.58 to −0.65, p’s < 0.05). Decreases in CSF cholinesterase activity were strongly associated with decreases in central adiposity, i.e., waist circumference (*r* = 0.92, *p* = 0.0002), android % fat (*r* = 0.74, *p* = 0.0082), and the android/gynoid ratio (*r* = 0.61, *p* = 0.048), as well as decreases in leg fat mass (*r* = 0.62, *p* = 0.044). Similarly, changes in the CSF AChE/BChE activity ratio were positively correlated with changes in leg fat mass (*r* = 0.64, *p* = 0.035) and inversely correlated with changes in VAT area (*r* = −0.64, *p* = 0.048) and VAT/SAT ratio (*r* = −0.72, *p* = 0.019). There was also a strong inverse correlation between changes in CSF neurogranin and changes in VAT area (*r* = −0.81, *p* = 0.015). Representative plots are shown in Supplementary Figure 2, which indicate an outlying data point. After removing this data point in sensitivity analyses, the correlations were attenuated (r’s = 0.42–0.69, p’s = 0.09–0.17).

## Discussion

The present study examined the effects of an MMK vs. AHA diet on body weight, body composition, and body fat distribution in older adults at risk for AD. We hypothesized that the MMK diet would lead to significant reductions in body weight and improvements in body composition and body fat distribution compared to the AHA diet and that these improvements would be associated with favorable changes in the CSF biomarker profile. We also hypothesized that there would be distinct associations between adiposity and CSF biomarkers depending on the location of fat (i.e., central/visceral vs. peripheral). We found that both the MMK and AHA diets led to similar improvements in adiposity. We also observed significant associations between changes in total and regional adiposity and changes in CSF biomarkers for both diets; yet, there were notable differences between diets. These findings provide novel evidence linking MMK diet-induced changes in neuropathology and adiposity, which could have important implications for the management of AD and related metabolic dysfunction.

Ketogenic diets induce a state of ketosis through the formation of ketone bodies, which serve as an alternative fuel source and provide robust neurological effects. The MMK diet was developed to improve adherence and reduce health risks associated with high saturated fat intake on a traditional ketogenic diet. Importantly, the MMK diet has comparable efficacy, but allows for slightly higher carbohydrate intake to permit increased consumption of fruits and vegetables, as well as fats and proteins derived from healthy sources such as olive oil and fish, consistent with a Mediterranean dietary pattern (Chianese et al., 2018). We previously reported that a 6-week MMK diet intervention led to significant improvements in CSF biomarkers, cerebral perfusion, memory, and metabolic indices in adults at risk for AD (Neth et al., 2020). In the present study, we extend these findings by demonstrating that the MMK diet is also associated with significant improvements in body weight, body composition and body fat distribution. Similar improvements in adiposity were observed following the AHA diet, although reductions in body weight was less compared to the MMK diet. The observed weight loss was not surprising given that caloric intake was reduced by ∼300–500 kcal/day (15–26%) while on the diets (Neth et al., 2020). Although the calorie content of the diets was matched to pre-study food intake, participants consumed less than the recommended amount of food during the intervention, a common occurrence with any prescribed dietary intervention. Importantly, the caloric intake did not differ between the two study diets, and so it is unlikely to have accounted for the different dietary effects. In response to both diets, there were significant decreases in total body fat due to reductions in all regions assessed including the trunk, legs, abdomen (android), and hips (gynoid), as well as VAT and SAT areas. There were also decreases in the trunk/leg and android/gynoid ratios suggesting improved body fat distribution. Taken together, these data suggest that in the presence of mild-to-moderate caloric restriction, both the MMK and AHA diets are effective at reducing body fat in older adults at risk for AD.

To our knowledge, this study is the first to report associations between adiposity and CSF AD biomarkers in response to a dietary intervention. Interestingly, we found that decreases in body fat on the MMK diet were mostly related to changes in CSF Aβ biomarkers, whereas decreases in body fat on the AHA diet were largely related to changes in CSF tau biomarkers. Modest fat loss on the MMK diet was associated with increased levels of CSF amyloid biomarkers (Aβ42, Aβ42/Aβ42 ratio, Aβ42/tau-p181 ratio), generally considered a more favorable profile, while greater fat loss was associated with worsened CSF Aβ profiles (i.e., decreased levels). On the other hand, fat loss on the AHA diet was associated with both decreases in CSF tau and tau-p181 (suggesting a favorable effect) and increases in the CSF tau-p181/tau ratio (suggesting an adverse effect). Improvements in cholinesterase activity on the AHA diet were also associated with greater fat loss, particularly in the abdominal region. Another striking difference between the diets was the opposing association observed between CSF neurogranin and VAT area following the MMK diet (i.e., positive correlation) and the AHA diet (i.e., negative correlation). While the reasons for these differences are not clear, the availability of carbohydrate vs. fat likely has important implications for both adipose and brain tissue and may modulate inter-organ relationships. With the sensitivity analyses identifying potential outliers in this small sample, it will be important for future studies to determine if the moderate-to-strong correlations observed in the present study hold up in a larger sample.

It is worth noting that some of the inconsistencies in the results may be related to the fact that the DXA-derived central fat measures used here (i.e., trunk fat, android fat), which are thought to reflect visceral adiposity, cannot truly distinguish VAT vs. SAT in those regions. The same is true for leg fat, which encompasses both SAT and VAT stored within and around skeletal muscle. This is important given the well-established anatomical, cellular, molecular, physiological, clinical and prognostic differences in fat depots (Lee et al., 2013). VAT is strongly associated with chronic inflammation, insulin resistance, and an increased risk for cardiometabolic diseases (Fox et al., 2007). In contrast, SAT may have a protective role by serving as a metabolic sink to store excess triglyceride during energy surplus, particularly when found in the hips and thighs. Indeed, studies show that abdominal VAT, thigh SAT, and thigh intermuscular fat are all independently associated with cardiometabolic risk (Snijder et al., 2005; Yim et al., 2008; Koster et al., 2010). In the present study, a higher VAT/SAT ratio at baseline was associated with higher neurogranin, NFL, sTREM2, and cholinesterase activity. These data suggest that VAT, either directly or indirectly *via* its association with cardiometabolic risk factors, may promote synaptic dysfunction, axonal injury, neuroinflammation and impaired neurotransmission. In contrast, higher total fat mass and peripheral adiposity, including higher leg and gynoid fat, were associated with lower NFL and/or cholinesterase activity, suggesting a protective role. Although none of the adiposity variables were significantly associated with CSF Aβ and CSF tau in cross-sectional analyses, there were trends (*p* < 0.10) for higher CSF tau-p181 and a lower CSF Aβ42/p-tau ratio in those with higher VAT. Despite the small sample sizes and multiple comparisons, our data suggest that the distribution of body fat in visceral vs. peripheral depots, rather than the total amount of fat, is linked to neuropathological processes in the brain. These findings are consistent with prior studies demonstrating a protective effective for higher BMI in late life (Atti et al., 2008; West and Haan, 2009) and suggest that higher body fat may reflect greater availability of lipids for fatty acid oxidation to use as an alternative fuel source in addition to glucose. More importantly, our novel findings indicate that in late life, greater brain neuropathology likely coincides with greater visceral adiposity and lower peripheral adiposity, independent of total body fat.

Our findings add to the growing body of evidence that body fat distribution provides useful information above and beyond BMI and waist circumference when considering obesity-related risks for AD. For example, abdominal VAT/SAT ratio was found to be ∼40% higher in AD patients compared to non-AD controls, despite similar BMI (Diehl-Wiesenecker et al., 2015). Several studies have also reported associations between higher abdominal VAT and adverse brain outcomes, including reduced total brain volume and hippocampal atrophy (Debette et al., 2010; Isaac et al., 2011), cerebral large and small vessel disease (Higuchi et al., 2017; Kim et al., 2017), reduced cortical thickness (Veit et al., 2014), impaired functional connectivity (Raschpichler et al., 2013), and microstructural damage (Widya et al., 2015). In addition, working memory is negatively associated with android fat and positively associated with gynoid fat in older adults (Forte et al., 2017). While the underlying mechanisms for these associations are not completely understood, adipose tissue-derived signals including adipokines, lipids, metabolites, non-coding RNAs, and exosomes likely play a role in modulating cross-talk between adipose tissue and the brain. These factors are secreted from adipose tissue and act as autocrine, paracrine, and endocrine mediators capable of modulating energy metabolism, inflammation, insulin signaling, neuronal function, and Aβ pathology (Kiliaan et al., 2014; Ishii and Iadecola, 2016). They may also underlie the metabolic and neurological benefits associated with a ketogenic diet (Giordano et al., 2014) and likely help explain the observed changes in the present study.

The limitations of this study include the small sample size, the lack of correction for multiple comparisons, and the brief intervention duration; however, this was a pilot study that was meant to be hypothesis generating and set the foundation for future studies to elucidate the role of adiposity in AD development and progression. A larger, well-powered clinical trial is needed to confirm the intervention effects and adequately test for associations between adiposity and CSF biomarkers. In addition, although DXA provides validated and reliable measures of body composition and body fat distribution, DXA-derived VAT is merely an estimate of visceral adiposity. Follow-up studies using more precise measures of body fat distribution from computed tomography scans will be necessary to further elucidate the role of fat in key VAT and SAT depots and are currently ongoing. Nevertheless, the present study is the first to explore associations between regional adiposity and CSF AD biomarkers and provides preliminary evidence that an MMK diet leads to favorable changes in both CSF biomarkers and total/regional adiposity. The finding that these changes were correlated suggest that in older adults at risk for AD, the MMK diet is a promising therapeutic intervention to improve brain health *via* changes in both central and peripheral metabolism, and that modest weight loss, while maximizing visceral fat loss and preserving peripheral fat depots, may have the greatest impact. Given that there are no known interventions that can preferentially target loss of specific fat depots, more research is needed to better understand the implications of weight and fat loss on neuropathological changes associated with the MMK diet.

## Data Availability Statement

The original contributions presented in the study are included in the article/Supplementary Material, further inquiries can be directed to the corresponding author.

## Ethics Statement

The studies involving human participants were reviewed and approved by the Wake Forest Institutional Review Board. The patients/participants provided their written informed consent to participate in this study.

## Author Contributions

TB and SC conceived the idea. BN helped design and execute the parent study. TR, HZ, and KB conducted the biomarker assays. TB drafted the manuscript. IL performed the data analysis. TB, IL, and SC interpreted the results. All authors reviewed and approved the final version of the manuscript.

## Conflict of Interest

HZ has served on scientific advisory boards and/or as a consultant for Abbvie, Alector, Annexon, Artery Therapeutics, AZTherapies, CogRx, Denali, Eisai, Nervgen, Novo Nordisk, Pinteon Therapeutics, Red Abbey Labs, Passage Bio, Roche, Samumed, Siemens Healthineers, Triplet Therapeutics, and Wave and has given lectures in symposia sponsored by Cellectricon, Fujirebio, Alzecure, Biogen, and Roche. KB has served as a consultant, on advisory boards, or on data monitoring committees for Abcam, Axon, BioArctic, Biogen, JOMDD/Shimadzu, Julius Clinical, Lilly, MagQu, Novartis, Ono Pharma, Pharmatrophix, Prothena, Roche Diagnostics, and Siemens Healthineers. HZ and KB are co-founders of Brain Biomarker Solutions in Gothenburg AB (BBS), which is a part of the GU Ventures Incubator Program, outside the work presented in this manuscript. The remaining authors declare that the research was conducted in the absence of any commercial or financial relationships that could be construed as a potential conflict of interest.

## Publisher’s Note

All claims expressed in this article are solely those of the authors and do not necessarily represent those of their affiliated organizations, or those of the publisher, the editors and the reviewers. Any product that may be evaluated in this article, or claim that may be made by its manufacturer, is not guaranteed or endorsed by the publisher.
