# Supplementary material for: Changes in Adiposity and Cerebrospinal Fluid Biomarkers Following a Modified Mediterranean Ketogenic Diet in Older Adults at Risk for Alzheimer’s Disease
Source: Front Neurosci. 2022 Jun 2;16:906539. doi: 10.3389/fnins.2022.906539 (PMC9202553; doi:10.3389/fnins.2022.906539)

**SUPPLEMENTARY MATERIAL**

Supplementary Table 1. CSF biomarker levels at baseline and after the MMK and AHA diets

Supplementary Figure 1. Changes in adiposity and CSF biomarkers on the MMK diet

Supplementary Figure 2. Changes in adiposity and CSF biomarkers on the AHA diet

|  | **Baseline** | | **Post-MMK** | | **Post-AHA** | |
| --- | --- | --- | --- | --- | --- | --- |
| **CSF Biomarker** | **N** | **Mean (SD)** | **N** | **Mean (SD)** | **N** | **Mean (SD)** |
| Aβ40 (pg/ml) | 18 | 10070 ± 5215 | 17 | 9790 ± 4519 | 14 | 7302 ± 4771 |
| Aβ42 (pg/ml) | 15 | 308 ± 67 | 15 | 348 ± 120 | 14 | 314 ± 106 |
| Aβ42/40 ratio | 15 | 0.04 ± 0.03 | 15 | 0.04 ± 0.02 | 14 | 0.06 ± 0.04 |
| tau (pg/ml) | 14 | 38 ± 26 | 15 | 32 ± 19 | 13 | 28 ± 20 |
| tau-p181 (pg/ml) | 15 | 35 ± 15 | 14 | 31 ± 10 | 14 | 34 ± 16 |
| tau-p181/tau ratio | 14 | 1.13 ± 0.50 | 15 | 1.21 ± 0.72 | 13 | 1.49 ± 0.57 |
| Aβ42/tau-p181 ratio | 15 | 9.78 ± 2.90 | 15 | 11.90 ± 4.63 | 14 | 10.11 ± 3.62 |
| NFL (ng/l) | 14 | 535 ± 247 | 14 | 507 ± 246 | 11 | 535 ± 213 |
| Neurogranin (pg/ml) | 12 | 110 ± 135 | 12 | 98 ± 120 | 9 | 119 ± 154 |
| YKL-40 (ng/ml) | 14 | 137 ± 40 | 14 | 139 ± 49 | 11 | 140 ± 53 |
| AChE (U/l) | 14 | 56 ± 13 | 14 | 49 ± 16 | 12 | 53 ± 13 |
| BChE (U/l) | 14 | 29 ± 7 | 14 | 27 ± 8 | 12 | 27 ± 7 |
| AChE/BChE | 14 | 1.94 ± 0.15 | 14 | 1.89 ± 0.46 | 12 | 1.96 ± 0.34 |
| sTREM2 (ng/ml) | 14 | 4.1 ± 2.1 | 14 | 3.9 ± 2.5 | 12 | 3.9 ± 2.5 |


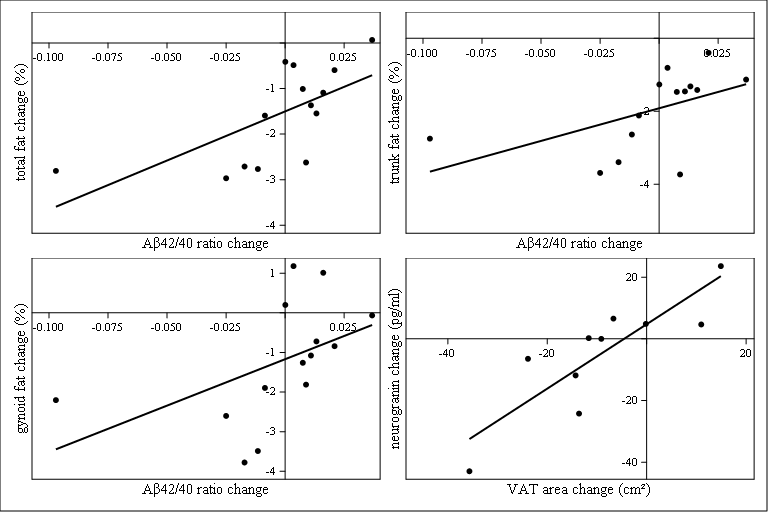


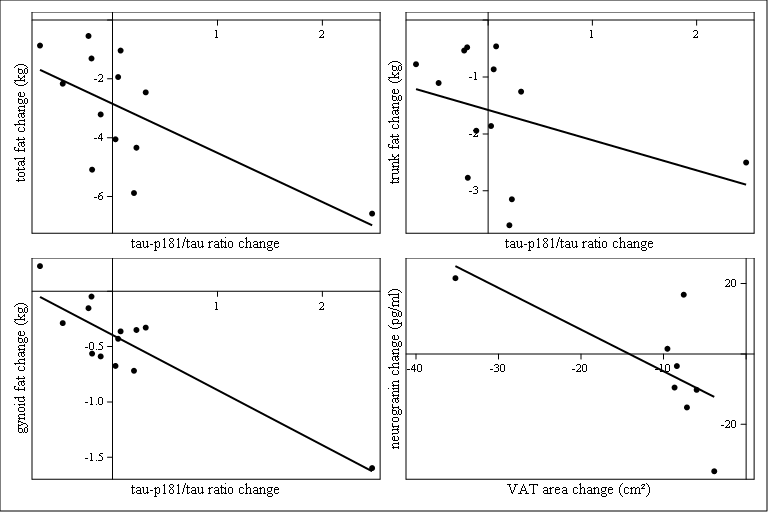

Supplement: Supplementary file 1 [file Data_Sheet_1.docx]
